# Improvement of Germination and Early Growth of Radish (*Raphanus sativus* L.) through Modulation of Seed Metabolic Processes

**DOI:** 10.3390/plants11060757

**Published:** 2022-03-12

**Authors:** Milica Kanjevac, Dragana Jakovljević, Marija Todorović, Milan Stanković, Svetlana Ćurčić, Biljana Bojović

**Affiliations:** 1 Department of Biology and Ecology, Faculty of Science, University of Kragujevac, 34000 Kragujevac, Serbia; milica.kanjevac@pmf.kg.ac.rs (M.K.); marija.stojadinov@pmf.kg.ac.rs (M.T.); mstankovic@kg.ac.rs (M.S.); biljana.bojovic@pmf.kg.ac.rs (B.B.); 2 Department of Natural Sciences, Faculty of Education, University of Kragujevac, 35000 Jagodina, Serbia; svetlana.curcic@pefja.kg.ac.rs

**Keywords:** priming, vegetable, MDA, phenolics compounds, flavonoids, DPPH

## Abstract

Radish (*Raphanus sativus* L.) is a vegetable cultivated worldwide because of its large succulent hypocotyls. The priming method initiates metabolic processes at early stages and regulates the metabolic events in seed necessary for germination. This research was conducted to examine the influence of various priming treatments on physiological performance (germination, growth, lipid peroxidation, primary and secondary metabolism) and antioxidant activity of radish seedlings. On the basis of germination and growth characteristics, vigor index, and relative water content in leaves, it was confirmed that priming treatments with 0.01% ascorbic acid (AA) and 1% KNO_3_ improves the initial stages of radish development. Furthermore, the efficiency of AA as a priming agent was confirmed through the reduction of malondialdehyde (MDA) level compared to unprimed seedlings. On the other hand, hormopriming with indole-3-acetic acid (IAA) significantly increased the concentration of photosynthetic pigments and total soluble leaf proteins compared to non-primed seedlings. The highest content of total phenolic compounds, including flavonoids, were obtained after hormopriming with 1 mM IAA and halopriming with 1% MgSO_4_. On the basis of the percentage of inhibition of DPPH radicals, it was confirmed that treatments with IAA and AA can improve the antioxidant activity of radish seedlings. This study provides useful information regarding the possibilities of pregerminative metabolic modulation through the seed priming for the biochemical and physiological improvement of radish, and this topic should be further investigated in order to determine the potential use of AA and IAA as suitable priming agents in radish commercial production.

## 1. Introduction

Sustainability and improvement of crop production is essential to meet consumer demands [1]. Seed germination and seedling growth are two critical points for the growth of any crop [2,3]. In order to provide high-quality seeds for successful agricultural production, various methods and techniques have been developed to improve the seed germination process, and among them, the priming method is one of the most effective since it improves seed performance and enables faster and synchronized germination [4,5]. On the basis of seed hydration, this method initiates metabolic processes in the early stages of germination but does not permit radicle protrusion. Activated metabolic processes are interrupted by placing the seeds on desiccation until they are placed again in a humid environment [5,6]. Major cellular processes are initiated as a consequence of rehydration, such as de novo ATP production, nucleic acid and protein synthesis, phospholipid and sterol accumulation, activation of antioxidant mechanisms, and DNA repair [7]. Consequently, primed seed shows an increased germination rate and a higher level of resistance to biotic and abiotic stress [8]. Depending on the type of plants and morphology and physiology of the seeds, different physicochemical and biological methods of treatment can be applied [9]. The most commonly used are hydro-, halo-, hormo-, osmo-, chemo-, and biotic treatments [10].

Due to their diverse beneficial properties for humans, crops such as radish are cultivated in large quantities. Radish (*Raphanus sativus* L.) is an essential root vegetable of the Brassicaceae family, cultivated as an annual horticultural crop and consumed worldwide due to its nutritional value [11,12]. On the basis of the season when crops are grown, radish varieties are classified into two groups: spring–summer and winter varieties [13]. Radish is cultivated mainly for its edible and fleshy roots. It is a good source of copper, potassium, calcium, magnesium, manganese, vitamin B6, and vitamin C [14]. In addition to containing protein, vitamins, and polysaccharides, radish root contains many phenolic compounds such as kaempferol, vanillin acid, cyanide, gentisic acid, hydrocinnamic acid, luteolin, myrcetin, and quercetin [15]. Aqueous extracts of radish root and leaves have shown antimutagenic and antimicrobial activities in vitro [15,16]. The antioxidant, antitumor, and antiviral activities of radish leaf extracts have also been confirmed [17]. However, it should be considered that radish, as a root vegetable, very easily accumulates a large amount of nitrate from the soil [18]. Accumulated nitrates in radish root can consequently represent a danger to human health [19].

In previous studies, radish breeding was performed in order to improve the ability to adapt to different growing conditions and resistance to pests [20]. In recent decades, changing consumer preferences have led to the replacement of previous breeding processes with new breeding methods. Great progress has been made by so-called red radish cultivars due to improved functionality [13]. According to Ashraf et al. [21], radish seed biopriming has a stimulating effect on growth physiology, which consequently affects the improvement of biochemical and antioxidant properties. Seed priming with zinc-chelated lysine (Zn-Lys) improved germination, yield, and nutritional quality of radish [22]. A significant effect of priming on the nutritional composition of radish was also noticed [23]. It has been previously confirmed that seed priming significantly helps plants to accelerate cell division, transport stored proteins, and accelerate seed germination [24].

Under the hypothesis that seed priming can improve the physiological performance of radish seedlings, we tested various priming agents and determined the impact of different priming treatments on germination characteristics, early growth, and development of radish, with the aim of expanding the possible uses of the tested priming agents. Considering the importance of radish and the efficiency of the priming technique, this study can provide an insight into the modulation of metabolic activities in radish seeds before radicle growth and can define the regulation of various processes by priming methods in radish, for which there is not a large amount of data in the literature.

## 2. Results

### 2.1. Seed Moisture Content

**Figure 1:**
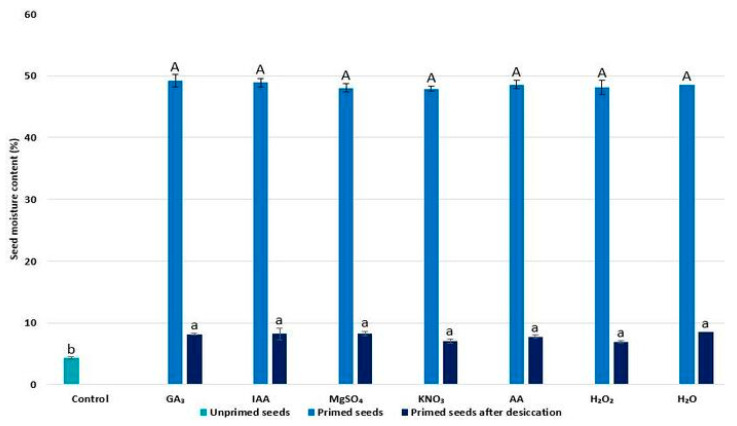
The moisture content of radish (*R*. *sativus*) seeds after priming for 24 h and after desiccation of primed seeds for 48 h prior to germination; control—unprimed seeds. The values represent the means of five replicates ± standard error. Small alphabetical letters (a,b) above the means show the differences (*p* ≤ 0.05) between primed seeds after desiccation and unprimed seeds, while capital alphabetical letters reveal the differences (*p* ≤ 0.05) between seeds immediately after 24 h of application of different priming agents by Tukey’s test.

To exclude the possibility that physiological performances of radish seedlings varied due to different moisture content in seeds after application of various priming agents, the seed moisture content was measured (both after priming for 24 h and after desiccation of primed seeds prior to germination) and compared with the control (unprimed seeds). The results are presented in Figure 1. The moisture content of unprimed radish seeds was 4.34%. The moisture content of seeds immediately after priming was nearly 10 times higher compared to the control seeds and tended to be similar, without significant differences between the priming agents applied. Additionally, the moisture content of seeds primed with various agents (after desiccation) was without significant differences among treatments applied. Still, the moisture content of primed seeds prior to germination was almost doubled compared to the control (unprimed) seeds.

### 2.2. Seed Germination Characteristics

The obtained results for the germination percentage (GP), mean germination time (MGT), and rate of germination (RG) are shown in Table 1. All applied treatments had stimulating effects on all the tested germination characteristics of radish seeds. The highest percentage of germination was recorded after treatment with AA (GP = 89.92%), which at the same time had a pronounced effect on germination rate. The most significant effect on mean germination time and rate of germination was achieved by H_2_O_2_ treatment (MTG = 1.50; RG = 67.12).

### 2.3. Growth Characteristics and Vigor Index

Influence of applied priming treatments on growth characteristics (shoot length, root length, fresh and dry weights) of radish are shown in Table 2. Compared to the control, the most significant effect on shoot length was observed after priming treatment with GA_3_ (shoot length = 5.73 cm). The most prominent effect on root length was observed after treatment with AA (root length = 10.85 cm), whereas for fresh and dry weights, the highest values were recorded after halopriming with KNO_3_ (0.0931 g and 0.0081 g, respectively).

Since increased vigor index values of seedlings can improve the initial stages of growth and development, the evaluation of vigor tests—seedling weight vigor index (SWVI), and seedling length vigor index (SWVI)—was performed. The results of these vigor tests (SLVI and SWVI) are presented in Table 2. On the basis of SLVI values, stimulating effects for each of the applied treatments can be seen, whereas the most significant effect was achieved with AA (SLVI = 1337.11). The results obtained for SWVI showed that the highest values were recorded after osmo- and halopriming without significant differences between these treatments.

### 2.4. Relative Water Content (RWC)

Leaf relative water content (RWC) varied depending on the treatment applied but without significant differences among applied treatments and unprimed seeds (Table 2). Still, after halopriming with KNO_3_, a slight increase in relative water was observed (RWC = 96.59%).

### 2.5. Concentration of Photosynthetic Pigments

Concentration of photosynthetic pigments in radish leaves depended on the applied priming treatment (Table 3). Observed through the concentration of total chlorophyll, chlorophyll a, and chlorophyll b, significant similarities were noticed in the synthesis of these photosynthetic pigments in relation to the applied priming treatment (Table 3). The highest concentration for all pigments was measured after seed priming with IAA, and the values were significantly different compared with both control and other treatments. The same trend was observed for the concentration of carotenoids. Oppositely, seed priming with H_2_O_2_ caused a significant decrease in the concentration of the examined photosynthetic pigments.

### 2.6. Concentration of Soluble Proteins

**Figure 2:**
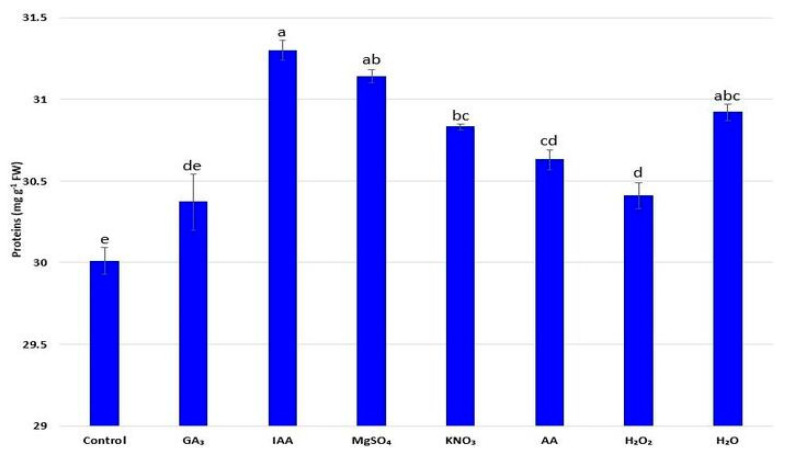
Influence of different priming treatments on the concentration of total soluble proteins in leaves of radish (*R. sativus*) seedlings; the values represent the means of six replicates ± standard error. Different letters indicate significant differences (*p* ≤ 0.05) between treatments according to Tukey’s test.

The influence of different priming agents on the concentration of total soluble proteins is shown in Figure 2, and it was noticed that seed priming had a stimulating effect on the concentration of soluble proteins. The highest content of total soluble proteins was measured during seed priming with IAA (31.30 mg g^−1^ FW).

### 2.7. Malondialdehyde Content (MDA)

**Figure 3:**
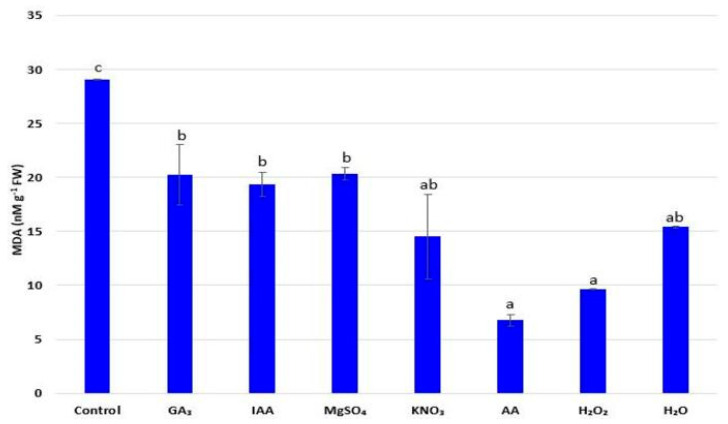
Influence of different priming treatments on malondialdehyde (MDA) content in leaves of radish (*R. sativus*) seedlings; the values represent the means of six replicates ± standard error. Different letters indicate significant differences (*p* ≤ 0.05) between treatments according to Tukey’s test.

MDA content is an important indicator of the degree of lipid peroxidation that causes cellular dysfunction. The examined radish seedlings showed significant differences in terms of MDA concentration depending on the applied treatment (Figure 3). All tested priming agents achieved positive effects since the MDA content was significantly lower after priming compared to seedlings estimated from nonprimed seeds. The obtained results indicate the ability of applied priming agents to reduce lipid peroxidation and increase the stability of cell membranes, with the most favorable effect observed after priming with AA (MDA content was about six time lower compared to unprimed seedlings). 

### 2.8. Concentration of Total Phenolic Compounds and Flavonoids

**Figure 4:**
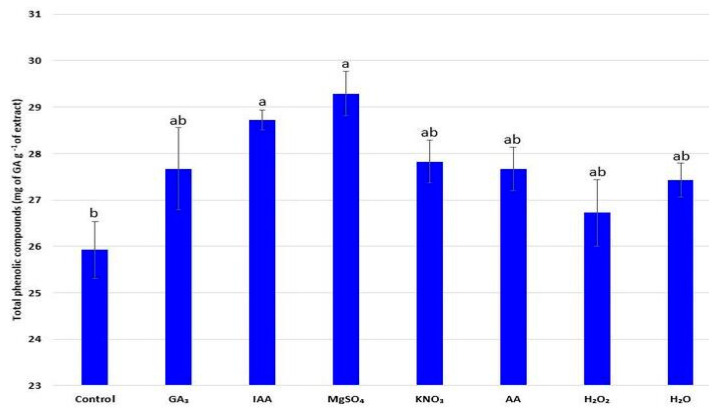
Influence of different priming treatments on concentration of total phenolic compounds (mg of GA g^−1^ of extract) in the aboveground part of radish (*R. sativus*) seedlings; the values represent the means of six replicates ± standard error. Different letters indicate significant differences (*p* ≤ 0.05) between treatments according to Tukey’s test.

The applied priming treatments had a significant effect on the synthesis of total phenolic compounds, as well as flavonoids. The measured concentrations of total phenolic compounds in radish extracts were in the range of 25.92 to 29.92 mg of GA g^−1^ of extract (Figure 4). All applied treatments induced synthesis of phenolic compounds, whereas the highest values were recorded after halopriming with MgSO_4_ and hormopriming with IAA (29.29 and 28.72 mg of GA g^−1^ of extract, respectively).

**Figure 5:**
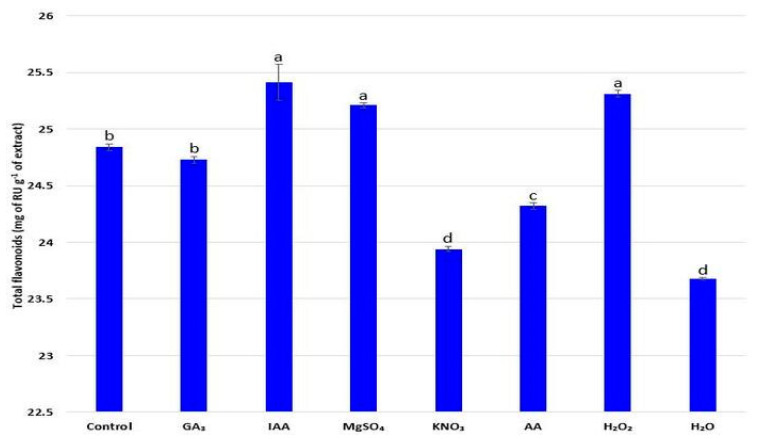
Influence of different priming treatments on concentration of flavonoids (mg of RU g^−1^ of extract) in the aboveground part of radish (*R. sativus*) seedlings; the values represent the means of six replicates ± standard error. Different letters indicate significant differences (*p* ≤ 0.05) between treatments according to Tukey’s test.

Flavonoid concentration in radish seedlings (ranging from 23.68 to 25.41 mg of RU g^−1^ of extract) was treatment-dependent (Figure 5). Increased synthesis of flavonoids was noticed after MgSO_4_, IAA, and H_2_O_2_ priming, with the highest values obtained after IAA hormopriming (25.41 mg of RU g^−1^ of extract). Significant decrease in flavonoid concentration was recorded after seed priming with KNO_3_, AA, and H_2_O.

### 2.9. Antioxidant Activity

The percentage of DPPH inhibition, determined spectrophotometrically through the activity of the radish extracts in the process of removing DPPH radicals, differed significantly depending on the concentration of the plant extract and the treatment applied (Table 4). Radish extracts were able to remove DPPH radicals, and the efficiency depended on the concentration, which can be explained by the dilution effect. The highest percentage of DPPH inhibition was observed for extracts obtained after seed priming with KNO_3_ (67.32% of DPPH inhibition for 500 μg mL^−1^ of plant extract) and was followed by AA (66.91% of DPPH inhibition for 500 μg mL^−1^ plant extract) and IAA treatments (66.81% of DPPH inhibition for 500 μg mL^−1^ plant extract). Between these treatments, obtained values were without significant differences. The lowest percentage of inhibition was recorded after treatment with H_2_O_2_ (61.69% of DPPH inhibition for 500 μg mL^−1^ plant extract).

## 3. Discussion

Radish, as a vegetable of great economic importance, contains essential nutrients for human nutrition and health [13], and cultivation of this vegetable is intensified worldwide. Seed germination is considered as an initial and critical determinant of crop success. Therefore, stimulation of seed germination can be used as a basic means to increase the yield of horticultural crops [25]. In this regard, seed priming is considered as an effective practice to improve overall seed germination and germination uniformity by using various methods and chemicals [26,27]. Seed priming can greatly improve the performance of radish seeds, which is of global economic importance.

Efficient, fast, and uniform seed germination is the first and most important step towards the success of any crop in commercial crop production [3]. If we consider the overall impact of the applied treatments on the germination characteristics, the results of this study showed remarkable improvement of radish germination process after seed priming. This could be related to the increase in moisture content in the seeds after priming [28]. The most pronounced effect on total germination was achieved with ascorbic acid (AA), while after H_2_O_2_ treatment, seeds germinated faster. According to Alves et al. [29], a positive effect of AA is as a result of its influence on numerous processes that improve the physiological activity of embryos and future seedlings. The positive effects of the application of H_2_O_2_ as a priming agent on germination dynamics have been confirmed previously [30,31]. Still, the dual role of hydrogen peroxide as a toxic molecule on one hand and as a signal molecule on the other should be considered [30], since in this study, H_2_O_2_ as a priming agent caused decrease of photosynthetic pigments, flavonoids, and total antioxidant activity. 

In addition to its improvement of germination characteristics, AA was the most significant priming agent for the improvement of radish growth, with an overall better stimulating effect on root elongation. Vitamins are compounds that have role of bioregulators and hormone precursors, and numerous physiological processes such as water absorption and cell division depend on vitamin availability [32]. AA is included in multiple enzymatic reactions, and it is essential to many aspects of plant growth including the regulation of cell cycle, cell division, and embryo development. The changes in ascorbic acid levels substantially alter the plant gene expression profile, raising the possibility of multiple reactions following alterations to AA content [33]. Improved root growth from seeds primed with ascorbic acid suggests that ascorbic acid is able to stimulate some cellular processes, including cell division and nutrient absorption in plant cells [34]. Similarly, a superior effect of KNO_3_ on radish mass was observed. Potassium nitrate, due to its nutritional value, in addition to acting as a salt, also has the property of a growth regulator, with a significant impact on plant mass [35]. Moaaz et al. [24] confirmed that KNO_3_ as a priming agent can significantly improve plant mass and growth. Moreover, a marked effect of these treatments in terms of vigor index was observed. Evaluation of seedling vigor index to determine seed quality is an important parameter since the seedling vigor index determines the viability of seeds as well as the ability of seeds to produce quality plants under conditions similar to those in nature [36]. High values of the seedling vigor index imply that plants will have the fastest possible germination, high percentage of germination, and higher seedling biomass and leaf area [37]. On the basis of the results of the seedling vigor index and early growth, it can be expected that the initial stages of growth and development of radish seeds will be better after priming with AA and halopriming with KNO_3_. 

Photosynthesis is a fundamental physiological process, and the parameters of photosynthesis are inextricably linked with the content of chloroplast pigments, primarily chlorophyll a, chlorophyll b, and carotenoids [38]. Low concentration of photosynthetic pigments can directly limit photosynthetic potential, and thus primary production [39]. Priming improves the concentration of photosynthetic pigments in radish leaves since seed priming resulted in a higher concentration of photosynthetic pigments compared to those developed from untreated seeds, whereas IAA treatment played a dominant role. IAA is known to stimulate plant growth, with a significant role in the process of photosynthesis and pigment formation [40]. The significant role of IAA in improving photosynthetic activity is also emphasized by Rhaman et al. [41]. Zhao et al. [42] confirmed that priming with IAA directly improved photosynthesis capacity, increasing stomatal conduction and intercellular CO_2_ concentration. The obtained results are in accordance with those from previous studies, where it was confirmed that priming agents under both favorable and unfavorable environmental conditions improve the concentration of photosynthetic pigments [43,44,45,46]. 

Secondary metabolites of phenolic nature, including flavonoids, are the main compounds responsible for the defense mechanism of plants [47], but also for the improved nutritional values of crops because of their well-known antioxidant activities. It has been previously confirmed that the concentration of phenolic compounds in radish depends on the particular radish variety and the influence of methods used to improve radish characteristics [13,48]. In this study, the use of hormone priming with IAA and osmopriming with MgSO_4_ had a superior effect in terms of total phenolic content and flavonoid concentration. The stimulatory effect of magnesium ions (Mg^2+^) on the synthesis of phenolic compounds was confirmed by Farzadfar et al. [49]. A similar observation on the multiple importance of auxin, including the effect on the biosynthesis of branched compounds such as phenolic compounds, were noted by Baliyan et al. [40]. Accordingly, it can be assumed that priming agents would play a key role in the defense mechanism of radish seedlings by increasing the concentration of total phenolic compounds including flavonoids. Furthermore, the positive effects of the named priming agents were observed in terms of protein content, as well as flavonoids. An increase in the total soluble protein content may be associated with improved physiological activities, mainly due to stimulation of the radish protein biosynthesis process [50].

Antioxidant compounds are naturally presented in plants and can be used preventively in the diet in order to prevent oxidative stress. Previous research has confirmed the antioxidant properties of radish [48,51]. Post-priming dehydration can lead to a number of cellular and biochemical processes, including activation of antioxidant defense systems [2], and in this study, seed priming with KNO_3_, AA, and IAA increased the ability of radish extracts to reduce free DPPH radicals, i.e., increased the total antioxidant activity of radish seedlings. According to Singhal et al. [52], the application of halopriming increases antioxidant capacity, probably acting as signaling molecules that drive several metabolic pathways, such as stomatal openings, abscisic acid production, synthesis, and increased antioxidant enzyme activity. Molecules of antioxidants such as ascorbic acid applied in the form of priming have been found to reduce and alleviate oxidative stress of plants to significant levels [53]. Madany et al. [54] confirmed that priming seeds with IAA has great potential to improve overall antioxidant capacity, especially at levels of oxidative stress. Similarly, Hamid et al. [55] and Ahmad et al. [56] documented that priming wheat and corn seeds resulted in an improved antioxidant defense system of their seedlings. Seed priming stimulates plants to better regulate oxidative stress, indicating the important role of priming in managing abiotic stress responses in crops. 

The regulatory effect of seed priming is closely related to the activation of the antioxidant protection system, the removal of reactive oxygen species (ROS), or the increased biosynthesis of phenolic compounds [3]. In addition, MDA content, as a final byproduct of lipid peroxidation, represents an indicative measure of oxidative damage [29]. In general, primed seed produced a lower content of MDA, which keeps the cell membrane stable and reduces ROS production by detoxifying them into non-toxic molecules, resulting in reduced oxidative stress and reduced membrane damage [52]. The effectiveness of AA priming agents in reducing ROS levels and the avoiding of lipid peroxidation was confirmed by Alves et al. [29]. In the defense of plants against oxidative stress, vitamins with their antioxidant properties play an important role as free radical scavengers. Ascorbic acid is affiliated with chloroplasts in which the effect of oxidative stress on photosynthesis is mitigated. AA is one of the non-enzymatic antioxidant compounds serving both as an electron donor to reduce the accumulation of ROS as well as the reaction substrate within the enzymatic cycle [32]. Auxins, especially IAA, can control the growth and development of plants. In addition to the well-known roles in plant elongation, apical dominance, or rhizogenesis, recent investigations suggest that auxin has influences on pigment and protein content, as well as increased concentrations of secondary metabolites where increased content of pigments under IAA treatments could be related to the protection of the leaf photosynthetic apparatus [57]. It is believed that the increase in antioxidant activity occurs when using endogenous IAA, where the hormone treatment stimulates plant secondary metabolism, thereby resulting in a higher concentration of secondary products such as phenols and flavonoids, which have a consequential impact on oxidative status in plants [58].

## 4. Materials and Methods

### 4.1. Experimental Design

Seeds of red radish (*Raphanus sativus* L. var. Saxa) were obtained from commercial sources (“Morpho d.o.o.” Belgrade). The seed preparation and priming method were conducted according to the work of Kanjevac et al. [59] with minor modifications. Applied treatments are presented in Table 5.

In brief, the surface-sterilization was performed with sodium hypochlorite, and sterilized seeds were primed with the appropriate treatment. For each treatment, 30 seeds were soaked in 10 mL of the appropriate solution for 24 h and then subjected to a desiccation process for 48 h at room temperature. Unprimed seeds were used as a control. Primed and unprimed seeds were placed in Petri dishes with filter paper (Whatman No. 1), soaked in 7 mL of distilled water, and incubated in a growth chamber (temperature 23 ± 2 °C, photoperiod 16/8 h, 60% humidity). For every treatment, germination was counted to the final count until no further germination occurred. The growth characteristics and physiological performances were evaluated 10 days after seed germination.

### 4.2. Seed Moisture Content

To estimate the moisture content of radish seeds during the different stages of seed priming, a low constant temperature oven method at 101–105 °C for 17 h was applied [60]. The same method was applied to measure the moisture content of unprimed seeds. In brief, seed moisture content was estimated for (1) seeds immediately after 24 h of application of different priming agents, (2) seeds after application of different priming agents followed by desiccation prior to germination, (3) unprimed seeds prior to germination. The seed moisture content (%) was calculated using the following equation:

$$\mathrm{Seed}\;\mathrm{moisture}\;\mathrm{content}=\frac{\mathrm{Weight}\;\mathrm{of}\;\mathrm{fresh}\;\mathrm{seeds}\;-\;\mathrm{Weight}\;\mathrm{of}\;\mathrm{dry}\;\mathrm{seed}}{\mathrm{Weight}\;\mathrm{of}\;\mathrm{fresh}\;\mathrm{seeds}}\times 100$$

### 4.3. Germination Characteristics

The germination characteristics of the tested species under the influence of different treatments were examined by measuring the total germination percentage (GP), mean germination time (MTG), and rate of germination (RG). Seeds were considered germinated after radicle appearance (at least 2 mm). Values were calculated according to the work of Bewley and Black [61] and Jakovljević et al. [62] on the basis of the following equations:

$$\mathrm{GP}=\frac{\mathrm{Total}\;\mathrm{seeds}\;\mathrm{germination}}{\mathrm{Total}\;\mathrm{number}\;\mathrm{of}\;\mathrm{planted}\;\mathrm{seeds}}\times 100\;\mathrm{MTG}=\frac{\sum^{\;}n_{i}\times {\;t}_{i}}{\sum^{\;}n_{i}};$$

n_i_ = number of newly germinated seeds;
t_i_ = days from start of the experiment to the observation.

RG=MGR ×100;

MGR = mean germination rate, the reciprocal of the mean germination time.

### 4.4. Growth Characteristics and Vigor Index

Index for evaluation of the seedling vigor was calculated according to the work of Bojović et al. [34] on the basis of the following equations:

Seedling Length Vigor Index (SLVI)=(Mean shoot length + Mean root length)× FGPSeedling Weight Vigor Index (SWVI)=Mean seedling weight × GPGP=Total germination percentage (%)

### 4.5. Relative Water Content

The leaf relative water content (RWC) is defined as the amount of water in plant leaves in relation to the state of full turgidity. The relative water content in the leaves (%) was measured and calculated according to the work of Dastborhan et al. [63] on the basis of the following equation:

$$\mathrm{RWC}=\frac{\mathrm{fresh}\;\mathrm{weitgh}-\mathrm{dry}\;\mathrm{weitgh}}{\mathrm{turgid}\;\mathrm{weitgh}-\mathrm{dry}\;\mathrm{weitgh}}\times 100$$

### 4.6. Measurement of Photosynthetic Pigments

The influence of the applied treatments on the concentration of photosynthetic pigments was examined by measuring the content of chlorophyll a (Chl a), chlorophyll b (Chl b), total chlorophyll (T-Chl, Chl a + b), and carotenoids (Cx + c). The preparation of plant material was performed according to the work of Bojović and Stojanović [64]. Briefly, 0.5 g of fresh leaf tissue was homogenized with the addition of 10 mL of 80% acetone and then centrifuged at 2500 rpm for 5 min. Concentrations of photosynthetic pigments were calculated according to the work of Wellburn [65] and expressed in relation to fresh weight (mg g^−1^ FW):

$$\mathrm{Chl}\;a+b=8.02\times A_{663}+20.20\times A_{646}\;\mathrm{Chl}\;a=12.21\times A_{663}-2.81\times A_{646}\;\mathrm{Chl}\;b=20.13\times A_{646}-5.03\times A_{663}\;C\;x+c=\left(1000\times A_{470}-3.27\times \mathrm{Chl}\;a-104\times \mathrm{Chl}\;b\right)÷198$$

### 4.7. Total Soluble Protein Concentration

The concentration of total soluble proteins in radish leaves was determined by the work of Lowry et al. [66] with bovine serum albumin (BSA) as standard. Values were calculated and expressed in relation to the fresh weight (mg g^−1^ FW).

### 4.8. Malondialdehyde Content

The determination of lipid peroxidation marker MDA was determined by the thiobarbituric acid (TBA) reaction in which mixture included enzyme solution, 0.5% TBA, and 20% trichloroacetic acid, while the absorbance was determined spectrophotometrically at 532 and 600 nm [67]. MDA content was expressed as nM g^−1^ FW.

### 4.9. Preparation of Plant Extracts

Juvenile plant parts (radish shoots) were sampled after 10 days from seed germination. The sampled plant material was dried at room temperature, at an air humidity of 55–60%, for 7 days. After that, the dry plant material was crushed to obtain plant powder, and plant extracts were prepared (20 mL of methanol for 1 g of plant powder). After 48 h, the extracted samples were filtered and then evaporated. To determine the concentration of total phenolic compounds and total flavonoids, as well as to measure the total antioxidant activity, a methanolic extract solution at a concentration of 1 mg/mL was used.

### 4.10. Determination of Total Phenolic Compounds

The amount of total phenolic compounds was determined according to the work of Singleton et al. [68] using gallic acid as a standard. Briefly, 0.5 mL of plant extract, 2 mL of 7.5 NaHCO_3_, and 2.5 mL of 10% Folin–Ciocalteu reagent were taken to prepare the reaction mixture. The samples were incubated for 15 min at 45 °C, and the absorbance was determined at a wavelength of 765 nm. The concentration of total phenols was calculated on the basis of the measured absorbance and calibration curve for gallic acid and was expressed in gallic acid equivalents (mg of GA g^−1^ of extract).

### 4.11. Determination of Total Flavonoids

The concentration of flavonoids was determined according to the work of Quettier-Deleuet al. [69] on the basis of the reaction of flavonoids with AlCl_3_, and rutin was used as a standard. The reaction mixtures were prepared with 1 mL of plant extract and 1 mL of 2% AlCl_3_ dissolved in methanol. The samples were incubated for one hour at room temperature, after which the absorbance was determined at a wavelength of 415 nm. Calculation was based on the measured absorbance and the calibration curve for rutin as a standard, and the concentration of flavonoids was expressed in rutin equivalents per milligram of extract (mg of RU g^−1^ of extract).

### 4.12. Evaluation of Antioxidant Activity

The total antioxidant activity was determined with the spectrophotometric method based on measuring the degree of neutralization of free 1,1-diphenyl-2-picrylhydrazyl (DPPH) radical [70]. For each sample, starting from the extract concentration of 1 mg/mL, dilution series were made to obtain concentrations of 500, 250, 125, 62.5, 31.25, 15.62, 7.81, 3.90, 1.99, and 0.97 μg mL^−1^. Diluted solutions (1 mL each) were mixed with 1 mL of DPPH radical solution (80 μg mL^−1^). The prepared samples were incubated for 30 min at room temperature in the dark, after which the absorbance was determined at a wavelength of 517 nm. The percentage inhibition of DPPH radicals was expressed on the basis of differences in the absorbance of samples of plant extracts and control samples (samples without extract solution):

$$\%\;\mathrm{inhibition}=\left(\frac{\;A\;\mathrm{of}\;\mathrm{control}-A\;\mathrm{of}\;\mathrm{sample}}{A\;\mathrm{of}\;\mathrm{control}}\;\right)\times 100$$

### 4.13. Statistical Analysis

The experiments were performed in at least five repetitions, and data are presented as mean ± SE (standard error). The program SPSS v.21 for Windows was used for the statistical evaluation of the results with an analysis of variance (ANOVA) and Tukey’s multiple range tests (*p* ≤ 0.05).

## 5. Conclusions

Seed priming is a promising tool for improvement of the radish seed germination process, as well as early seedling growth. Radish responded positively to all applied priming treatments, which showed different efficacy depending on the tested characteristics. It was observed that seed germination, radish growth performance, and level of malondialdehyde were improved by priming with AA. In addition, a significant influence of applied priming agents on the physiological processes of seedlings was shown, whereas hormopriming with IAA improved the concentration of photosynthetic pigments and proteins, as well as total phenolic content and flavonoid concentration. At the same time, priming with IAA was effective in the improvement of total antioxidant activity compared to unprimed radish seedlings. Because the seed priming technique is a simple, fast, inexpensive method and less labor-intensive than conventional strategies, the agricultural industry can easily adopt it. Further studies should include an assessment of radish growth under adverse conditions to show whether the priming method could improve the production of this valuable crop under diverse cultivation conditions.

## Figures and Tables

**Table 1:** Effect of different priming treatments on germination percentage (%) (GP), mean time for germination (MTG), and rate of germination (RG) of radish (*R. sativus*).

Treatment	GP	MTG	RG
Control	65.56 ± 5.88 ^#^c	2.73 ± 0.17 c	36.90 ± 2.28 c
GA_3_	82.22 ± 2.94 ab	1.65 ± 0.16 ab	61.69 ± 6.24 ab
IAA	68.51 ± 1.49 bc	2.37 ± 0.11 bc	42.36 ± 1.85 bc
MgSO_4_	84.45 ± 2.22 a	2.06 ± 0.05 abc	48.68 ± 1.21 abc
KNO_3_	87.78 ± 2.22 a	1.69 ± 0.06 ab	59.44 ± 2.13 abc
AA	89.92 ± 3.78 a	1.91 ± 0.34 ab	55.75 ± 9.81 abc
H_2_O_2_	82.22 ± 2.22 ab	1.50 ± 0.10 a	67.12 ± 4.36 a
H_2_O	81.11 ± 2.94 abc	1.89 ± 0.14 ab	53.58 ± 3.95 abc

^#^ The values represent the means of six replicates ± standard error. Different letters indicate significant differences (*p* ≤ 0.05) between treatments according to Tukey’s test.

**Table 2:** Effect of different priming treatments on shoot and root length (cm), fresh and dry weight (g), leaf relative water content—RWC (%), seedling weight vigor index (SWVI), and seedling length vigor index (SWVI) of radish (*R. sativus*).

Treatment	Root Length	Shoot Length	Fresh Weitgh	Dry Weight	SLVI	SWVI	RWC
Control	7.26 ± 0.64 ^#^b	4.74 ± 0.16 ab	0.0818 ± 0.00 ab	0.0061 ± 0.00 b	786.68 ± 70.56 e	5.36 ± 0.48 d	92.57 ± 0.55 a
GA_3_	9.30 ± 0.67 ab	5.73 ± 0.29 a	0.0639 ± 0.00 b	0.0067 ± 0.00 ab	1235.82 ± 44.18 ab	5.25 ± 0.19 d	89.09 ± 2.62 a
IAA	8.19 ± 0.57 ab	5.20 ± 0.20 ab	0.0832 ± 0.01 ab	0.0077 ± 0.00 ab	917.30 ± 20.00 ce	5.70 ± 0.13 cd	90.54 ± 0.18 a
MgSO_4_	8.65 ± 0.73 ab	4.64 ± 0.35 ab	0.0899 ± 0.01 a	0.0073 ± 0.00 ab	1122.29 ± 29.55 bc	7.59 ± 0.20 a	93.82 ± 1.00 a
KNO_3_	7.82 ± 0.68 b	4.74 ± 0.24 ab	0.0931 ± 0.00 a	0.0081 ± 0.00 a	1102.47 ± 27.93 bc	8.17 ± 0.21 a	96.59 ± 1.17 a
AA	10.85 ± 0.76 a	4.25 ± 0.25 b	0.0777 ± 0.00 ab	0.0075 ± 0.00 ab	1357.79 ± 57.11 a	6.98 ± 0.29 ab	92.33 ± 2.09 a
H_2_O_2_	7.13 ± 0.55 b	4.45 ± 0.24 b	0.0846 ± 0.01 ab	0.0074 ± 0.00 ab	952.15 ± 25.75 cde	6.96 ± 0.19 abc	94.82 ± 1.20 a
H_2_O	9.34 ± 0.64 ab	4.56 ± 0.22 b	0.0740 ± 0.00 ab	0.0064 ± 0.00 ab	1127.47 ± 40.87 bd	6.00 ± 0.22 bd	93.32 ± 2.79 a

^#^ The values represent the means of six replicates ± standard error. Different letters indicate significant differences (*p* ≤ 0.05) between treatments according to Tukey’s test.

**Table 3:** Effect of different priming treatments on pigments content (mg g^−1^ FW) in leaves of radish (*R. sativus*) seedlings.

Treatment	Total Chlorophyll	Chlorophyll *a*	Chlorophyll *b*	Carotenoids
Control	0.953 ± 0.007 ^#^cd	0.464 ± 0.007 d	0.366 ± 0.001 b	0.083 ± 0.0009 f
GA_3_	0.779 ± 0.006 f	0.427 ± 0.002 e	0.253 ± 0.003 g	0.098 ± 0.0006 d
IAA	1.235 ± 0.003 a	0.665 ± 0.001 a	0.413 ± 0.002 a	0.119 ± 0.0003 a
MgSO_4_	0.958 ± 0.002 c	0.521 ± 0.001 c	0.315 ± 0.001 d	0.106 ± 0.0003 b
KNO_3_	1.070 ± 0.003 b	0.588 ± 0.001 b	0.345 ± 0.002 c	0.101 ± 0.0003 c
AA	0.936 ± 0.002 d	0.514 ± 0.001 c	0.303 ± 0.001 e	0.104 ± 0.0006 bc
H_2_O_2_	0636 ± 0.003 g	0.358 ± 0.001 f	0.198 ± 0.002 h	0.070 ± 0.0007 g
H_2_O	0.805 ± 0.003 e	0.440 ± 0.001 e	0.263 ± 0.002 f	0.090 ± 0.0006 e

^#^ The values represent the means of six replicates ± standard error. Different letters indicate significant differences (*p* ≤ 0.05) between treatments according to Tukey’s test.

**Table 4:** Influence of different priming treatments on percentage of DPPH inhibition in the aboveground parts of radish (*R. sativus*) seedlings.

Extract Concentration (μg mL^−1^)	Treatment							
	Control	GA_3_	IAA	MgSO_4_	KNO_3_	AA	H_2_O_2_	H_2_O
500	64.96 ± 0.24 ^#^b	64.45 ± 0.06 b	66.81 ± 0.59 a	62.30 ± 0.12 c	67.32 ± 0.18 a	66.91 ± 0.65 a	61.69 ± 0.12 c	65.98 ± 0.01 ab
250	43.24 ± 1.54 a	42.63 ± 0.59 a	45.80 ± 0.06 a	44.67 ± 6.27 a	48.16 ± 1.78 a	51.64 ± 2.48 a	42.93 ± 0.06 a	46.72 ± 1.89 a
125	25.62 ± 0.36 abc	19.26 ± 3.32 c	22.44 ± 2.90 bc	25.41 ± 0.35 abc	30.23 ± 2.90 ab	32.89 ± 2.07 a	24.90 ± 0.06 abc	26.85 ± 1.78 abc
62.5	16.29 ± 1.48 a	14.96 ± 1.89 a	17.52 ± 1.13 a	15.57 ± 0.12 a	19.16 ± 0.06 a	19.78 ± 0.06 a	16.60 ± 0.95 a	16.80 ± 1.42 a
31.25	12.09 ± 1.54 a	11.89 ± 1.65 a	12.50 ± 1.89 a	12.19 ± 1.95 a	13.01 ± 1.00 a	13.73 ± 1.06 a	12.81 ± 1.24 a	11.68 ± 1.89 a
15.62	11.27 ± 1.30 a	10.76 ± 2.19 a	10.66 ± 1.78 a	10.86 ± 2.13 a	10.15 ± 1.24 a	10.45 ± 1.30 a	11.48 ± 1.54 a	10.56 ± 2.07 a
7.81	10.56 ± 1.12 a	9.73 ± 1.71 a	9.63 ± 2.13 a	9.53 ± 2.19 a	8.92 ± 1.60 a	9.74 ± 1.60 a	10.86 ± 1.42 a	9.84 ± 1.78 a
3.9	9.74 ± 1.36 a	9.22 ± 1.89 a	9.02 ± 2.13 a	9.02 ± 2.25 a	8.61 ± 1.54 a	9.02 ± 1.89 a	9.53 ± 1.13 a	9.53 ± 1.71 a
1.9	9.02 ± 1.07 a	8.51 ± 2.07 a	8.61 ± 2.25 a	8.71 ± 2.31 a	8.10 ± 1.72 a	8.51 ± 2.07 a	8.92 ± 1.24 a	8.20 ± 1.89 a
0.97	8.72 ± 1.00 a	7.89 ± 1.95 a	8.30 ± 2.19 a	8.30 ± 2.43 a	7.07 ± 1.24 a	8.20 ± 2.13 a	9.12 ± 1.71 a	7.38 ± 2.13 a

^#^ The values represent the means of six replicates ± standard error. Different letters indicate significant differences (*p* ≤ 0.05) between treatments according to Tukey’s test.

**Table 5:** Summary of the applied priming treatments.

Priming Treatments	Concentration
Gibberellic acid—GA_3_	1 mM
Indol-3-acetic acid—IAA	
MgSO_4_	1%
KNO_3_	
Hydrogen peroxide—H_2_O_2_	1%
Ascorbic acid—AA	0.01%
H_2_O	-

## Data Availability

The data presented in this study are available on request from the corresponding author.

## References

[1] Colla G., Rouphael Y. (2015). Biostimulants in horticulture. Sci. Hortic..

[2] Ibrahim E.A. (2016). Seed priming to alleviate salinity stress in germinating seeds. J. Plant Physiol..

[3] Zulfiqar F. (2021). Effect of seed priming on horticultural crops. Sci. Hortic..

[4] Tanou G., Fotopoulos V., Molassiotis A. (2012). Priming against environmental challenges and proteomics in plants: Update and agricultural perspectives. Front. Plant Sci..

[5] Mamun A.A., Naher U.A., Ali M.Y. (2018). Effect of seed priming on seed germination and seedling growth of modern rice (*Oryza sativa* L.) varieties. Agriculturists.

[6] Ashraf M., Foolad M.R. (2005). Pre-sowing seed treatment—A shotgun approach to improve germination, plant growth, and crop yield under saline and non-saline conditions. Adv. Agron..

[7] Galland M., Huguet R., Arc E., Cueff G., Job D., Rajjou L. (2014). Dynamic proteomics emphasizes the importance of selective mRNA translation and protein turnover during *Arabidopsis* seed germination. Mol. Cell. Proteom..

[8] Jisha K.C., Vijayakumari K., Puthur J.T. (2013). Seed priming for abiotic stress tolerance: An overview. Acta Physiol. Plant..

[9] Yadav P.V., Maya K., Zakwan A. (2011). Seed priming mediated germination improvement and tolerance to subsequent exposure to cold and salt stress in capsicum. Res. J. Seed Sci..

[10] Paparella S., Araújo S.S., Rossi G., Wijayasinghe M., Carbonera D., Balestrazzi A. (2015). Seed priming: State of the art and new perspectives. Plant Cell Rep..

[11] Xie Y., Xu L., Wang Y., Fan L., Chen Y., Tang M., Luo X., Liu L. (2018). Comparative proteomic analysis provides insight into a complex regulatory network of taproot formation in radish (*Raphanus sativus* L.). Hortic. Res..

[12] Muleke E.M.M., Wang Y., Zhang W.T., Liang X.U., Ying J.L., Karanja B.K., Zhu X.W., Fan L.X., Ahmadzai Z., Liu L.W. (2021). Genome-wide identification and expression profiling of MYB transcription factor genes in radish (*Raphanus sativus* L.). J. Integr. Agric..

[13] Park C.H., Baskar T.B., Park S.Y., Kim S.J., Valan Arasu M., Al-Dhabi N.A., Kim J.K., Park S.U. (2016). Metabolic profiling and antioxidant assay of metabolites from three radish cultivars (*Raphanus sativus*). Molecules.

[14] Gamba M., Asllanaj E., Raguindin P.F., Glisic M., Franco O.H., Minder B., Bussler W., Metzger B., Kern H., Muka T. (2021). Nutritional and phytochemical characterization of radish (*Raphanus sativus*): A systematic review. Trends Food Sci. Technol..

[15] Jakmatakul R., Suttisri R., Tengamnuay P. (2009). Evaluation of antityrosinase and antioxidant activities of *Raphanus sativus* root: Comparison between freeze-dried juice and methanolic extract. Thai J. Pharm. Sci..

[16] Beevi S.S., Mangamoori L.N., Dhand V., Ramakrishna D.S. (2009). Isothiocyanate profile and selective antibacterial activity of root, stem, and leaf extracts derived from *Raphanus sativus* L.. Foodborne Path. Dis..

[17] Umamaheswari A., Prabu S.L., John S.A., Puratchikody A. (2021). Green synthesis of zinc oxide nanoparticles using leaf extracts of *Raphanus sativus var. Longipinnatus* and evaluation of their anticancer property in A549 cell lines. Biotechnol. Rep..

[18] Kerckhoffs H., Zhang L. (2021). Application of Central Composite Design on Assessment and Optimization of Ammonium/Nitrate and Potassium for Hydroponically grown Radish (*Raphanus sativus*). Sci. Hortic..

[19] Kiani A., Sharafi K., Omer A.K., Matin B.K., Davoodi R., Mansouri B., Sharafi H., Soleimani H., Massahi T., Ahmadi E. (2022). Accumulation and human health risk assessment of nitrate in vegetables irrigated with different irrigation water sources-transfer evaluation of nitrate from soil to vegetables. Environ. Res..

[20] Pei Y., Yao N., He L., Deng D., Li W., Zhang W. (2019). Comparative study of the morphological, physiological and molecular characteristics between diploid and tetraploid radish (*Raphunas sativus* L.). Sci. Hortic..

[21] Ashraf R., Sultana B., Riaz S., Mushtaq M., Iqbal M., Nazir A., Atif M., Zafar Z. (2018). Fortification of phenolics, antioxidant activities and biochemical attributes of radish root by plant leaf extract seed priming. Biocatal. Agric. Biotechnol..

[22] Noman A., Ali Q., Maqsood J., Iqbal N., Javed M.T., Rasool N., Naseem J. (2018). Deciphering physio-biochemical, yield, and nutritional quality attributes of water-stressed radish (*Raphanus sativus* L.) plants grown from Zn-Lys primed seeds. Chemosphere.

[23] Baenas N., Villaño D., García-Viguera C., Moreno D.A. (2016). Optimizing elicitation and seed priming to enrich broccoli and radish sprouts in glucosinolates. Food Chem..

[24] Moaaz A.M., Javed T., Mauro R.P., Shabbir R., Afzal I., Yousef A.F. (2020). Effect of seed priming with potassium nitrate on the performance of tomato. Agriculture.

[25] Demir I., Ozden E., Yıldırım K.C., Sahin O., Van Staden J. (2018). Priming with smoke-derived karrikinolide enhances germination and transplant quality of immature and mature pepper seed lots. S. Afr. J. Bot..

[26] Adhikari B., Adhikari M., Ghimire B., Adhikari B.C., Park G., Choi E.H. (2020). Cold plasma seed priming modulates growth, redox homeostasis and stress response by inducing reactive species in tomato (*Solanum lycopersicum*). Free Radic. Biol. Med..

[27] Karim M., Sani M., Hossain N., Uddain J., Azad M.O.K., Kabir M., Rahman M.S., Choi K.Y.N., Naznin M.T. (2020). Stimulatory Effect of Seed Priming as Pretreatment Factors on Germination and Yield Performance of Yard Long Bean (*Vigna unguiculata*). Horticulturae.

[28] Badek B., van Duijn B., Grzesik M. (2006). Effects of water supply methods and seed moisture content on germination of *China aster* (*Callistephus chinensis*) and tomato (*Lycopersicon esculentun* Mill.) seeds. Eur. J. Agron..

[29] Alves R.D.C., Rossatto D.R., da Silva J.D.S., Checchio M.V., de Oliveira K.R., Oliveira F.D.A., de Queiroz S.F., Cruz M.C.P., Gratão P.L. (2021). Seed priming with ascorbic acid enhances salt tolerance in micro-tom tomato plants by modifying the antioxidant defense system components. Biocatal. Agric. Biotechnol..

[30] Wojtyla Ł., Lechowska K., Kubala S., Garnczarska M. (2016). Different modes of hydrogen peroxide action during seed germination. Front. Plant Sci..

[31] Miladinov Z., Balešević-Tubić S., Đukić V., Ilić A., Čobanović L., Dozet G., Merkulov-Popadić L. (2018). Effect of priming on soybean seed germination parameters. Acta Agric. Serb..

[32] Gaafar A.A., Ali S.I., El-Shawadfy M.A., Salama A.Z., Sekara A., Ulrich C., Abdelhamid M.T. (2020). Ascorbic acid induces the increase of secondary metabolites, antioxidant activity, growth, and productivity of the common bean under water stress conditions. Plants.

[33] Gallie D. (2013). L-Ascorbic acid: A multifunctional molecule supporting plant growth and development. Scientifica.

[34] Salemi F., Esfahani M.N., Tran L.S.P. (2019). Mechanistic insights into enhanced tolerance of early growth of alfalfa (*Medicago sativa* L.) under low water potential by seed-priming with ascorbic acid or polyethylene glycol solution. Ind. Crop. Prod..

[35] Anwar A., Xianchang Y.U., Yansu L.I. (2020). Seed priming as a promising technique to improve growth, chlorophyll, photosynthesis and nutrient contents in cucumber seedlings. Not. Bot. Horti Agrobot. Cluj-Napoca.

[36] Bojović B.M., Jakovljević D.Z., Ćurcić S.S., Stanković M.S. (2018). Phytotoxic potential of common nettle (*Urtica dioica* L.) on germination and early growth of cereals an vegetables. Allelopath. J..

[37] Schuch L.O.B., Nedel J.L., de Assis F.N., Maia M.D.S. (2000). Seed vigour and growth analysis of black oats. Sci. Agric..

[38] Gengmao Z., Yu H., Xing S., Shihui L., Quanmei S., Changhai W. (2015). Salinity stress increases secondary metabolites and enzyme activity in safflower. Ind. Crop. Prod..

[39] Anjum S.A., Xie X.Y., Wang L.C., Saleem M.F., Man C., Lei W. (2011). Morphological, physiological and biochemical responses of plants to drought stress. Afr. J. Agric. Res..

[40] Baliyan N., Dhiman S., Dheeman S., Kumar S., Maheshwari D.K. (2021). Optimization of indole-3-acetic acid using response surface methodology and its effect on vegetative growth of chickpea. Rhizosphere.

[41] Rhaman M.S., Imran S., Rauf F., Khatun M., Baskin C.C., Murata Y., Hasanuzzaman M. (2021). Seed priming with phytohormones: An effective approach for the mitigation of abiotic stress. Plants.

[42] Zhao T., Deng X., Xiao Q., Han Y., Zhu S., Chen J. (2020). IAA priming improves the germination and seedling growth in cotton (*Gossypium hirsutum* L.) via regulating the endogenous phytohormones and enhancing the sucrose metabolism. Ind. Crop. Prod..

[43] Al-Amri S.M. (2013). Improved growth, productivity and quality of tomato (*Solanum lycopersicum* L.) plants through application of shikimic acid. Saudi J. Biol. Sci..

[44] Yan M. (2015). Seed priming stimulate germination and early seedling growth of *Chinese cabbage* under drought stress. S. Afr. J. Bot..

[45] Piri R., Moradi A., Balouchi H., Salehi A. (2019). Improvement of cumin (*Cuminum cyminum*) seed performance under drought stress by seed coating and biopriming. Sci. Hortic..

[46] Valivand M., Amooaghaie R., Ahadi A. (2019). Seed priming with H_2_S and Ca^2+^ trigger signal memory that induces cross-adaptation against nickel stress in zucchini seedlings. Plant Physiol. Biochem..

[47] Mousavi A., Pourakbar L., Moghaddam S.S., Popović-Djordjević J. (2021). The effect of the exogenous application of EDTA and maleic acid on tolerance, phenolic compounds, and cadmium phytoremediation by okra (*Abelmoschus esculentus* L.) exposed to Cd stress. J. Environ. Chem. Eng..

[48] Goyeneche R., Roura S., Ponce A., Vega-Gálvez A., Quispe-Fuentes I., Uribe E., Di Scala K. (2015). Chemical characterization and antioxidant capacity of red radish (*Raphanus sativus* L.) leaves and roots. J. Funct. Foods..

[49] Farzadfar S., Zarinkamar F., Hojati M. (2017). Magnesium and manganese affect photosynthesis, essential oil composition and phenolic compounds of *Tanacetum parthenium*. Plant Physiol. Biochem..

[50] Hatami M., Khanizadeh P., Bovand F., Aghaee A. (2021). Silicon nanoparticle-mediated seed priming and *Pseudomonas* spp. inoculation augment growth, physiology and antioxidant metabolic status in *Melissa officinalis* L. plants. Ind. Crop. Prod..

[51] Takaya Y., Kondo Y., Furukawa T., Niwa M. (2003). Antioxidant constituents of radish sprout (kaiware-daikon), *Raphanus sativus* L.. J. Agric. Food Chem..

[52] Singhal R.K., Pandey S., Bose B. (2021). Seed priming with Mg(NO_3_)_2_ and ZnSO_4_ salts triggers physio-biochemical and antioxidant defense to induce water stress adaptation in wheat (*Triticum aestivum* L.). Plant Stress.

[53] Elkelish A., Qari S.H., Mazrou Y.S., Abdelaal K.A., Hafez Y.M., Abu-Elsaoud A.M., Batiha G.E.S., El Esawi M., El Nahhas N. (2020). Exogenous ascorbic acid induced chilling tolerance in tomato plants through modulating metabolism, osmolytes, antioxidants, and transcriptional regulation of catalase and heat shock proteins. Plants.

[54] Madany M.M., Zinta G., Abuelsoud W., Hozzein W.N., Selim S., Asard H., Abd Elgawad H. (2020). Hormonal seed-priming improves tomato resistance against broomrape infection. J. Plant Physiol..

[55] Hamid M., Ashraf M.Y., Arashad M. (2008). Influence of salicylic acid seed priming on growth and some biochemical attributes in wheat grown under saline conditions. Pak. J. Bot..

[56] Ahmad I., Ahmad T.K.A., Basra S.M., Hasnain Z., Ali A. (2012). Effect of seed priming with ascorbic acid, salicylic acid and hydrogen peroxide on emergence, vigor and antioxidant activities of maize. Afr. J. Biotechnol..

[57] Esan A.M., Olaiya C.O., Omolekan T.O., Aremu K.A., Adeyemi H.R.Y. (2020). Comparative effects of indole acetic acid and on photosynthetic pigments and mineral contents of two genotypes of okra under salinity sress. Eur. J. Agric. Food Sci..

[58] Li Y.P., Yu C.X., Qiao J., Zang Y.M., Xiang Y., Ren G.X., Wang L., Zhang X.Y., Liu C.S. (2016). Effect of exogenous phytohormones treatment on glycyrrhizic acid accumulation and preliminary exploration of the chemical control network based on glycyrrhizic acid in root of *Glycyrrhiza uralensis*. Rev. Bras. Farmacogn..

[59] Kanjevac M., Jakovljević D., Bojović B. (2021). Improvement of physiological performance of selected cereals by modulating pregerminative metabolic activity in seeds. Cereal Res. Commun..

[60] Hanson J. (1985). Procedures for Handling Seeds in Genebanks.

[61] Bewley J.D., Black M. (1994). Seeds: Physiology of Development and Germination.

[62] Jakovljević D., Bojović B., Stanković M., Topuzović M. (2020). Characteristics of in vitro seed germination of three basil genotypes under different nutrition. Kragujev. J. Sci..

[63] Dastborhan S., Ghassemi-Golezani K. (2015). Influence of seed priming and water stress on selected physiological traits of borage. Folia Hortic..

[64] Bojović B.M., Stojanović J. (2005). Chlorophyll and carotenoid content in wheat cultivars as a function of mineral nutrition. Arch. Biol. Sci..

[65] Wellburn A.R. (1994). The spectral determination of chlorophylls a and b, as well as total carotenoids, using various solvents with spectrophotometers of different resolution. J. Plant Physiol..

[66] Lowry O.H., Rosebrough N.J., Farr A.L., Randall R.J. (1951). Protein measurement with the Folin phenol reagent. J. Biol. Chem..

[67] Jakovljević D., Topuzović M., Stanković M. (2019). Nutrient limitation as a tool for the induction of secondary metabolites with antioxidant activity in basil cultivars. Ind. Crop. Prod..

[68] Singleton V.L., Orthofer R., Lamuela-Raventós R.M. (1999). Analysis of total phenols and other oxidation substrates and antioxidants by means of folin-ciocalteu reagent. Meth. Enzymol..

[69] Quettier-Deleu C., Gressier B., Vasseur J., Dine T., Brunet C., Luyckx M., Cazin M., Cazin J.K., Bailleul F., Trotin F. (2000). Phenolic compounds and antioxidant activities of buckwheat (*Fagopyrum esculentum* Moench) hulls and flour. J. Ethnopharmacol..

[70] Stanković M.S., Radić Z.S., Blanco-Salas J., Vázquez-Pardo F.M., Ruiz-Téllez T. (2017). Screening of selected species from Spanish flora as a source of bioactive substances. Ind. Crop. Prod..

